# Percutaneous operative treatment of fragility fractures of the pelvis may not increase the general rate of complications compared to non-operative treatment

**DOI:** 10.1007/s00068-021-01660-w

**Published:** 2021-04-03

**Authors:** Laura Gericke, Annemarie Fritz, Georg Osterhoff, Christoph Josten, Philipp Pieroh, Andreas Höch

**Affiliations:** Department of Orthopedics, Trauma and Plastic Surgery, University Hospital Leipzig, University of Leipzig, Liebigstrasse 20, 04103 Leipzig, Germany

**Keywords:** Fragility fractures of the pelvis, Non-operative treatment, Operative treatment, Complications, Mortality

## Abstract

**Purpose:**

Despite an increasing number of fragility fractures of the pelvis (FFP) over the last 2 decades, controversy persists on their therapy with special regard to potential complications. Therefore, the present study compared the complication rates and in-hospital mortality of non-operative therapy, percutaneous treatment and open reduction and internal fixation (ORIF) of pelvic fractures in elderly patients.

**Methods:**

All consecutive patients treated for FFP between January 2013 and December 2017 aged 65 years or older were retrospectively identified from an institutional database. Demographic data and specific patient data were collected with a special focus on pre-existing comorbidities. General and surgical complications, hospital length of stay (LOS) and mortality rates were compared.

**Results:**

379 patients (81.3 ± 7.5 years; 81% female) were identified, 211 (55.7%) were treated non-operatively, 74 (19.5%) percutaneously and 94 (24.8%) with ORIF. The rate of general complications did not differ between treatment groups (non-operative: 21.8%; percutaneous: 28.4%; ORIF: 33.0%; *p* = 0.103). Surgery-related complications were twofold more frequent in the ORIF group as than in the percutaneously treated group (18.1% vs. 9.5%). The LOS differed significantly (non-operatively: 8.9 ± 7.1 days; percutaneous: 16.6 ± 8.2 days; ORIF: 19.3 ± 12.8 days; *p* < 0.001). Hospital mortality rate was higher in patients with ORIF (5.3%) than percutaneous treatment (0%) (*p* = 0.044).

**Conclusions:**

Complication rates and hospital mortality in elderly patients with FFPs are high and associated with long LOS. For surgical treatment of FFPs, the complication rate and mortality can be significantly reduced using percutaneous procedures compared to ORIF. Therefore, percutaneous surgery should be preferred where possible.

**Supplementary Information:**

The online version contains supplementary material available at 10.1007/s00068-021-01660-w.

## Introduction

Despite an increasing number of fragility fractures of the pelvis (FFP) over the last 2 decades [1–3], controversy persists on their appropriate treatment with special regard to potential complications. Independent of the treatment, the main goal for these fractures is to reduce pain and thus accelerate patient`s mobilization [4–6]. In several cases non-operative treatment including adequate pain therapy, physical therapy and osteoporosis therapy fulfills these aims [7–9].

In case of unstable fractures or in case of failure of non-operative treatment, surgical stabilization is recommended [6]. In recent years, various procedures have been developed or adopted from conventional pelvic surgery for young patients [10–13]. Percutaneous procedures are increasingly used in the treatment of FFP in old and frequently comorbid patients. In contrast to young patients, the focus is not necessarily on anatomical reconstruction, but on stabilization and the resulting pain relief and fracture healing [14].

Complication rates in elderly patients with a pelvic fracture can be up to 58% even with non-operative treatment of supposed simple fractures [8, 15]. Data on complication rates after operative treatment of FFP are only available in restricted numbers and are usually limited to case series of specific surgical techniques [16, 17].

Therefore, in this study we compared the complication rates and in-hospital mortality of non-operative therapy and percutaneous surgery and open reduction and internal fixation (ORIF).

## Patients and methods

The present study was approved by the local ethics committee (151/17-ek).

### Patients

**Fig. 1:**
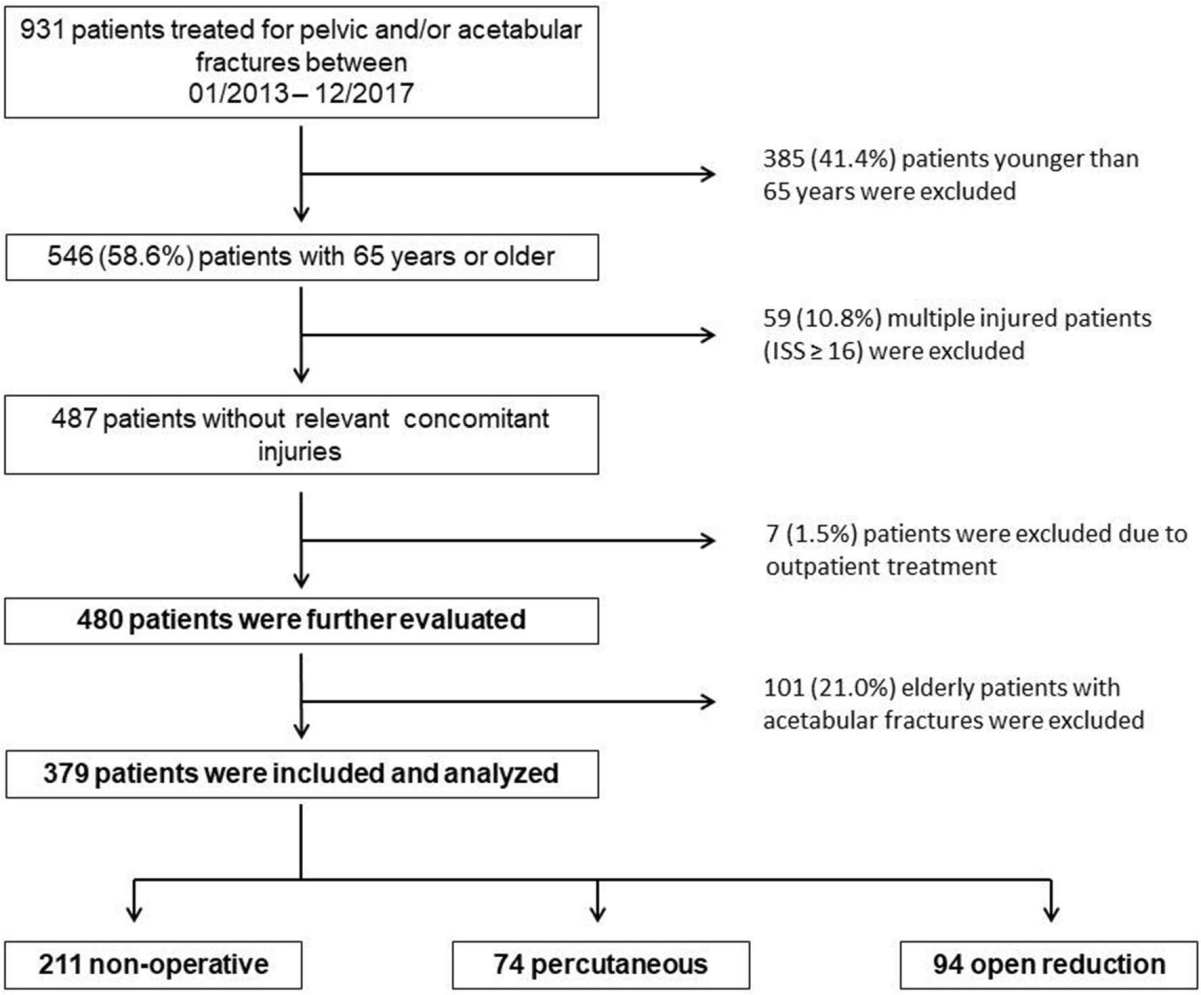
Study protocol. Patients treated for fragility fractures of the pelvis between 01/2013 and 12/2017 with complete data and aged ≥ 65 years were evaluated. Patients with multiple injuries and patients treated solely in the outpatient clinic were excluded (ISS = Injury Severity Score)

All consecutive patients aged 65 years or older treated for FFP were retrospectively identified from an institutional database. According to the inclusion and exclusion criteria, 379 patients were available for evaluation (Fig. 1).

### Treatment

Patients were divided into three groups depending on their treatment: non-operative treatment, percutaneous surgery, and ORIF. Percutaneous surgery was defined as the use of iliosacral/transsacral screws (augmentation was used depending to the surgeon), pubic ramus screws and supraacetabular screws. ORIF included all open approaches (modified Stoppa approach or first window of the ilioinguinal approach) or their combination followed by internal fixation by plates or screws. Spinopelvic fixation was classified as an open method in this study. If only a part of the surgery was performed open, it was considered to belong to the ORIF group.

According to our institutional protocol, FFP I and FFP II were managed non-operatively using pain killers and mobilization under physiotherapeutic supervision. In case mobilization failed within 4–5 days, percutaneous surgical treatment or ORIF was performed. FFP III and FFP IV with corresponding symptoms were indicated for surgical therapy if there were no contraindications or the patient refused surgery. All patients were allowed full weight-bearing postoperatively, regardless of treatment.

### Data acquisition

Patient`s demographics and the following comorbidities were recorded: diabetes mellitus; arterial hypertension; chronic heart failure (CHF); chronic obstructive pulmonary disease (COPD); osteoporosis; renal insufficiency; systemic malignancy; and dementia.

All fractures were classified according to Rommens and Hofmann with FFP classification and retrospectively verified by the senior author [6].

The following documented acute major complications that required specific treatment were evaluated: pneumonia, acute cardiac event, thromboembolism, stroke, multiple organ failure, systemic inflammatory response syndrome (SIRS), sepsis, and delirium. Treated urinary tract infections (UTI) were not counted as major complications, as there are concerns about the documentation and definition of the attending clinician.

Surgical complications documented include the following: unexpected intraoperative bleeding, iatrogenic nerve damage, implant malpositioning, surgical site infections, and implant failure with loss of reduction.

In addition, the time to surgery, length of hospital stay (LOS) and mortality were recorded and evaluated.

### Statistical analysis

Statistical analyses were performed in SPSS 24.0 (SPSS Inc., Chicago, IL, USA). Unless otherwise denoted, data were summarized as mean with standard deviation (SD).

Primary outcome was the occurrence of acute complications during the hospitalization. The hypothesis was that the complication rate as well as LOS and mortality depend on the extent of the surgical therapy. In order to assess possible interfering factors, the baseline characteristics and pre-existing conditions were compared between non-operative, percutaneous and ORIF groups. For all analyzed data, a normal distribution could be determined using the Shapiro–Wilk test. Nominal data were compared with the Chi-square test and for continuous data the Kruskal–Wallis test and Mann–Whitney *U* test were used depending on the number of compared groups. The level of significance was defined as *p* < 0.05.

## Results

Of 379 patients included (age: 81.3 ± 7.5 years; 81% female), 211 (55.7%) were treated non-operatively, 74 (19.5%) percutaneously and 94 (24.8%) with ORIF. Patient’s demographics and their comparison in relation to their treatment group are presented in Table 1. A significant difference of age distribution was found between the treatment groups with oldest patient in non-operative group and the youngest in the ORIF group (*p* < 0.001).

**Table 1:** Baseline data compared between treatment groups

	Total *n* = 379	Treatment			*p*
		Non-operative *n* = 211	Percutaneous *n* = 74	ORIF *n* = 94	
Age [years (mean ± SD)]	81.3 ± 7.5	82.8 ± 7.8	80.4 ± 6.2	78.7 ± 6.9	< 0.001
Gender [*n* (%)]					
Female	305 (80.5)	175 (82.9)	60 (81.1)	70 (74.5)	> 0.2
Male	74 (19.5)	36 (17.1)	14 (18.9)	24 (25.5)	
Comorbidity [*n* (%)]					
None	9 (2.4)	9 (4.3)	0 (0.0)	0 (0.0)	< 0.01
≤ 2	141 (37.4)	81 (38.4)	21 (28.4)	39 (41.5)	
> 2	227 (59.9)	121 (57.3)	53 (71.6)	53 (56.4)	

Almost all patients had pre-existing comorbidities (97.6%); significantly different between the groups was the incidence of dementia that correlated with non-operative treatment (Table 2).

**Table 2:** Pre-existing comorbidities compared between treatment groups. Several comorbidities per patient are possible

Comorbidities [*n* (%)]	Total	Treatment			*p*
		Non-operative	Percutaneous	ORIF	
None	9 (2.4)	9 (4.3)	0 (0.0)	0 (0.0)	0.002
Diabetes mellitus	92 (24.3)	52 (24.6)	20 (27.0)	20 (21.3)	0.722
Arterial hypertension	315 (83.1)	177 (83.9)	64 (86.5)	74 (78.7)	0.550
Osteoporosis	143 (37.7)	71 (33.6)	36 (48.6)	36 (38.3)	0.080
Renal insufficiency	47 (12.4)	22 (10.4)	13 (17.6)	12 (12.8)	0.280
COPD	52 (13.7)	28 (13.3)	10 (13.5)	14 (14.9)	0.906
Chronic heart failure	86 (22.7)	42 (19.9)	23 (31.1)	21 (22.3)	0.155
Systemic malignancy	35 (9.2)	17 (8.1)	5 (6.8)	13 (13.8)	0.176
Dementia	61 (16.1)	46 (21.8)	5 (6.8)	10 (10.6)	0.003

*COPD* chronic obstructive pulmonary disease

**Fig. 2:**
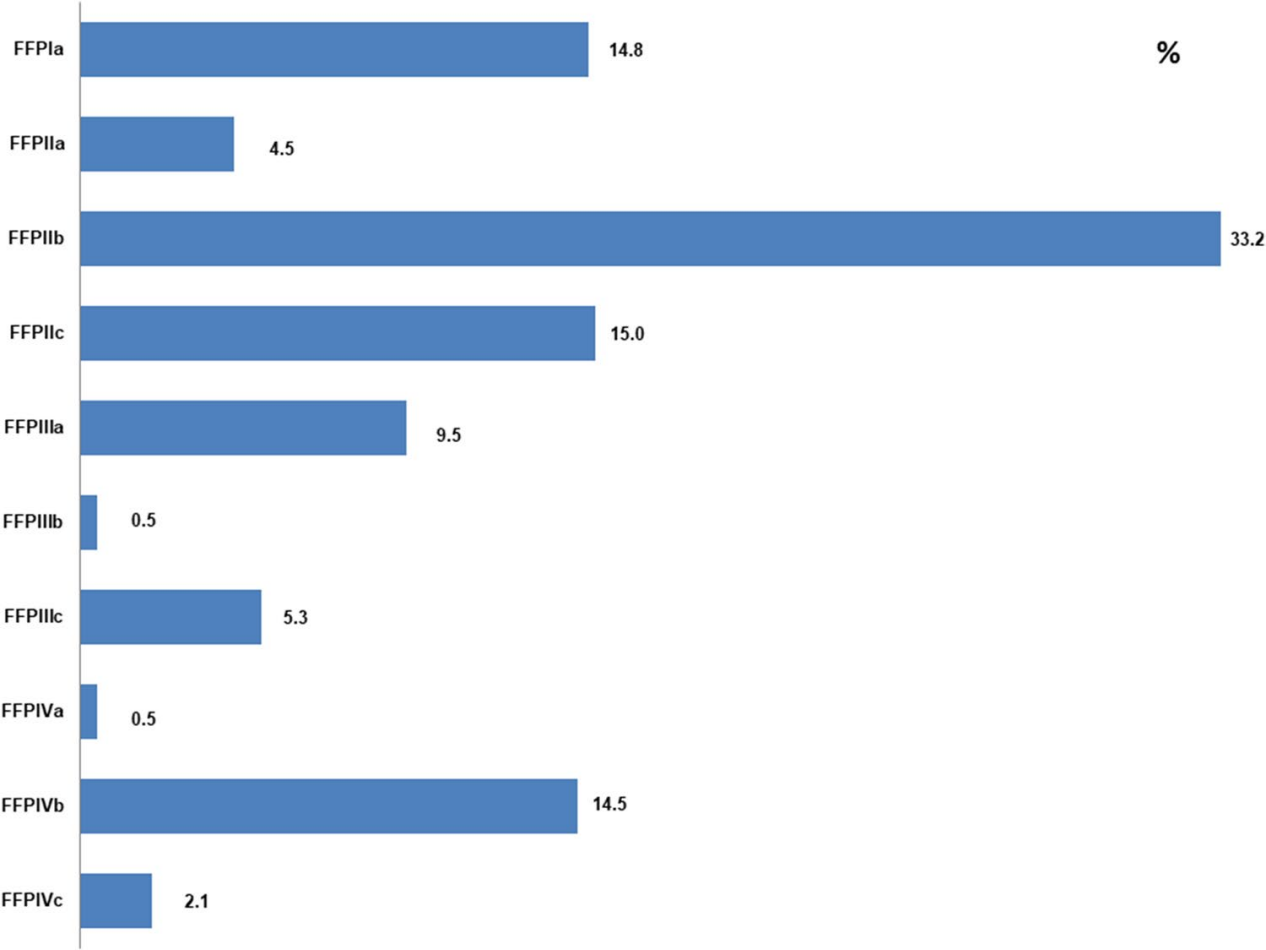
Fracture distribution of the fragility fractures of the pelvis according to Rommens and Hofmann [6]

**Fig. 3:**
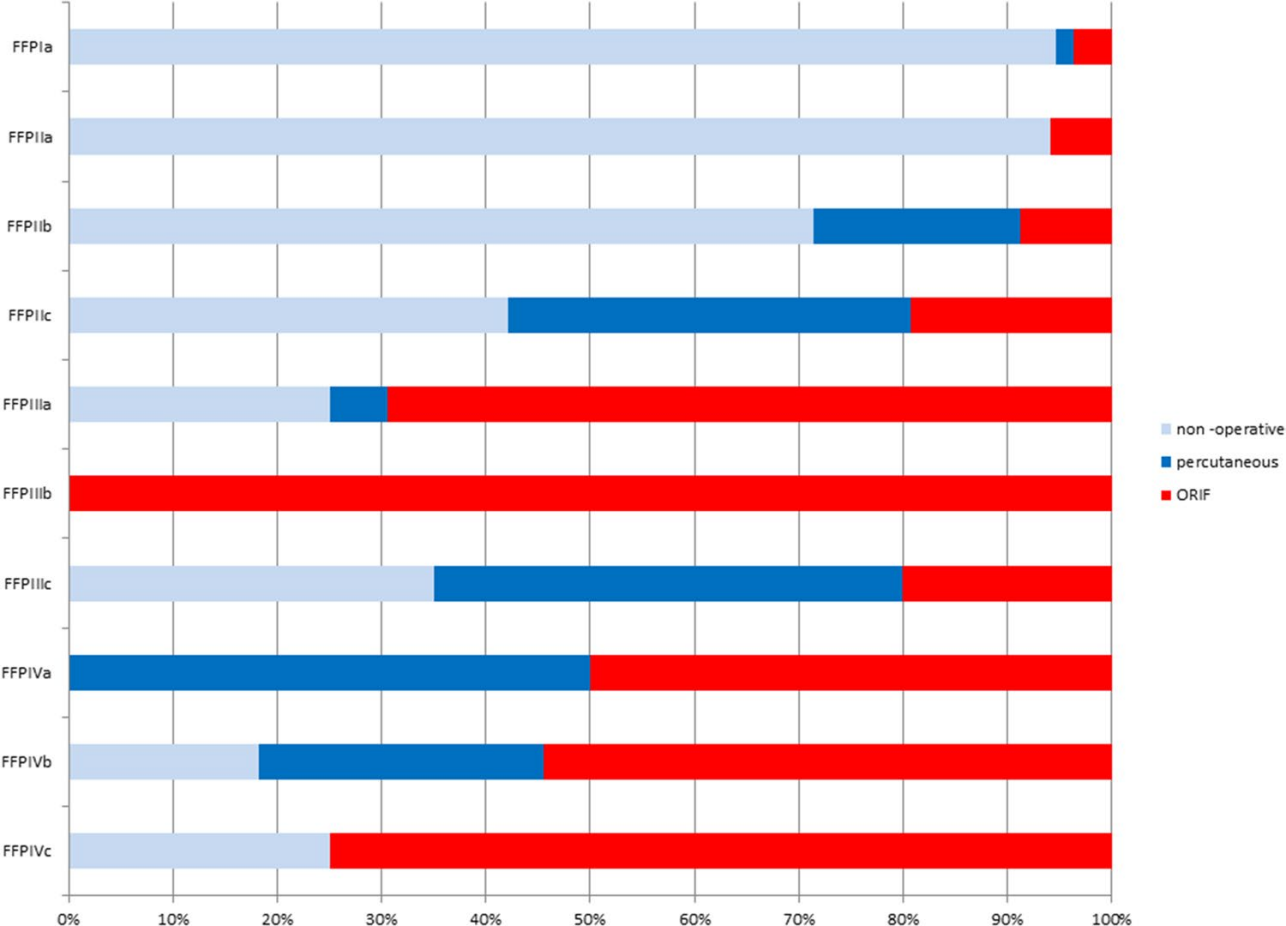
Type and frequency of treatment depending on FFP classification

The distribution of fractures and the treatment of the respective fracture types are shown in Figs. 2 and 3.

Summarizing the general complications including UTIs and comparing them yielded non-significant differences between treatment groups (non-operative: 21.8%; percutaneous: 28.4%; ORIF: 33.0%; *p* = 0.103) (Table 3).

**Table 3:** General complications. Several complications per patient are possible

Complications [*n* (%)]	Total	Treatment			*p*
		Non-operative	Percutaneous	ORIF	
Overall	36 (9.5)	22 (10.4)	3 (4.1)	11 (11.7)	0.193
Multiple organ failure	9 (2.4)	5 (2.4)	1 (1.4)	3 (3.2)	0.739
Pneumonia	13 (3.4)	7 (3.3)	2 (2.7)	4 (4.3)	0.852
Delirium	9 (2.4)	6 (2.8)	0 (0)	3 (3.2)	0.321
Cardiac event	4 (1.1)	2 (0.9)	0 (0)	2 (2.1)	0.397
SIRS/sepsis	3 (0.8)	1 (0.5)	0 (0)	2 (2.1)	0.223
Pulmonary embolism	1 (0.3)	1 (0.5)	0 (0)	0 (0)	0.671
Thrombosis	1 (0.3)	1 (0.5)	0 (0)	0 (0)	0.671

*SIRS* systemic inflammatory response syndrome

Surgery-related complications occurred in a total of 15.7% of patients and had a twofold higher incidence in the ORIF group compared to the percutaneously treated group (18.1% vs. 9.5%; *p* = 0.113). Predominant is the difference in the infection rate (12.8% vs. 0%; *p* < 0.001). In 10/12 patients, the implant was preserved after revision surgery due to infection, two patients received a change from a spinopelvic fixation to sacroiliac screw fixation. Details on the complications are given in Table 4.

**Table 4:** Surgery-related complications depending on the technique of surgery

Complications [*n* (%)]	Total *n* = 168	Treatment		*p*
		percutaneous *n* = 74	ORIF *n* = 94	
Overall	24 (15.7)	7 (9.5)	17 (18.1)	0.113
Malpositioning	5 (3.0)	5 (6.8)	1 (1.1)	0.190
Bleeding	3 (1.8)	1 (1.4)	2 (2.1)	0.666
Infection	12 (7.1)	–	12 (12.8)	< 0.001
Loss of reduction	2 (1.2)	1 (1.4)	1 (1.1)	> 0.05
Nerve damage	2 (1.2)	–	2 (2.1)	> 0.05

Several complications per patient are possible

The shortest LOS was observed in non-operatively treated patients (8.9 ± 7.1 days), followed by the percutaneously treated (16.6 ± 8.2 days) and the longest remaining were patients with ORIF (19.3 ± 12.8 days) (*p* < 0.001). Preoperative waiting time for percutaneous treated patients was longer than for ORIF (6.9 ± 5.0 days vs. 5.0 ± 3.8 days; *p* < 0.007), but postoperative hospital stay was distinctly shorter for percutaneously treated patients than for ORIF (9.7 ± 6.0 days vs. 14.2 ± 12.3 days; *p* < 0.001).

Total hospital mortality rate was 2.9% and there was no difference between non-operative (2.8%) and operative treatment (3.0%) (*p* = 1.0) overall. Nevertheless, mortality was significantly higher for ORIF (5.3%) compared to percutaneous treatment (0.0%) (*p* = 0.044).

A detailed data overview depending on the FFP classification and treatment group are presented in the supplemental Table 1.

## Discussion

The aim of this study was to compare the general complication rates of non-operative therapy, percutaneous treatment and ORIF of FFPs in patients aged over 65 years.

Overall, the complication rate and mortality in our investigated patient population is very high and the treatment is associated with long LOS especially in operative-treated patients. The complication rate increased once again in the patients treated operatively. However, the examination of patients treated percutaneously showed no higher complication rate than in non-operatively treated patients and a significantly lower complication rate than in patients with ORIF.

The general complication rate of 9.5% was found to coincide with previously published data, which may vary significantly depending on the detection of urinary tract infections [8, 15, 18, 19]. For this reason, we analyzed complications without urinary tract infections. Loggers was able to show that, in contrast to purely ventral fractures, the complication rate increases significantly from 18 to 44% when the posterior pelvic ring is involved [20]. Although UTIs were included, this rate was higher than in our study.

Surgical complications were found in 15.7% of all patients. The most common surgical complication was infection, which was only seen after ORIF in 12.8% of cases. Ochenjele et al. reported similar rates of complications (overall 15%; infections 8%). However, younger and multiple injured patients were included from Ochenjele et al. limiting the comparison [21]. Data on elderly patients with fragility FFP are limited to case series reporting on specific percutaneous surgical techniques [13, 22].

As previously published, we also found a prolonged inpatient stay with a maximum for ORIF of almost 20 days on average [4, 16]. From the clinical experience, this can be explained partly by the time-consuming perioperative management and the organization of a mostly necessary rehabilitation or nursing care after discharge.

The in-hospital mortality rate of 2.9% also corresponds to previous reports (1.3–7.6%) [16, 23, 24]. This high complication rate in isolated pelvic fractures, which does not usually result from high-energy accidents in old patients, indicate a very fragile patient population with high risk factors [25].

In a recent registry study “general health” was frequently stated as a reason for non-operative treatment and thus influences the surgeons decision [25]. The present study results suggest that dementia leads more often to non-operative treatment. Reasons for this may be an expected higher mortality and complication rate [26, 27]. In addition, the clinical assessment of patients with dementia is significantly more difficult, especially with regard to pain. Apart from this factor, no difference could be found between the therapy groups with regard to comorbidities.

Several limitations to the present study must be stated. Due to the retrospective nature of the study, no statement about the treatment decision is possible. Certainly, there is a bias in this examination, as the decision on therapy also depends on comorbidities in individual cases. It is well known that besides chronological age, biological age and activity level of the patients affect the therapy decisions. To be able to compare this more objectively, a validated frailty index should be used in prospective studies.

Not every patient can be treated non-operatively due to persistent pain and immobilization. As is well known, persistent immobilization increases the risk of other complications such as thrombosis, pneumonia, pressure ulcerations, etc. [16, 28–30]. The complication rate of percutaneous treatment is not significantly higher compared to non-operative therapy and other studies have already shown significant pain reduction with such techniques [13, 22, 31]. Especially for FFP, percutaneous solutions for surgical treatment of almost all fracture types are possible. In elderly patients, the goal of surgical therapy is not necessarily anatomical reduction, but rather stabilization of the fracture to induce and facilitate a healing process and alleviate the patients' pain [4, 5, 14, 25].

Another weakness of this study is the lack of follow-up after discharge. Especially for FFP, we know that it can be a creeping process and a fracture successfully treated non-operatively at the beginning can also worsen and require surgical treatment later on [5, 17, 32].

## Conclusion

Inpatient complication rates in elderly patients with FFP are high and associated with prolonged LOS.

In case of operative treatment of FFP in elderly patients with existing comorbidities, the complication rate and mortality can be significantly reduced using percutaneous procedures instead of ORIF. Therefore, a percutaneous procedure should be preferred whenever possible.

## Supplementary Information

Below is the link to the electronic supplementary material.

Supplementary file1 (DOCX 14 KB)

Supplementary file2 (DOCX 27 KB)
