# Supplementary material for: Percutaneous operative treatment of fragility fractures of the pelvis may not increase the general rate of complications compared to non-operative treatment
Source: Eur J Trauma Emerg Surg. 2021 Apr 3;48(5):3729–35. doi: 10.1007/s00068-021-01660-w (PMC9532300; doi:10.1007/s00068-021-01660-w)
Supplement: Supplementary file 1 — Supplementary file1 (DOCX 14 KB) [file 68_2021_1660_MOESM1_ESM.docx]

**Supplemental table 1** Overview of procedures used for surgical treatment.

| Percutaneous treatment (n=74) | | ORIF (n=94) | |
| --- | --- | --- | --- |
| Posterior pelvic ring | Anterior pelvic ring | Posterior pelvic ring | Anterior pelvic ring |
| 87 in screw augmented transiliosacral screws  17 transiliosacral screws without augmentation  5 transsacral screws  3 supraacetabular screw fixations | 41 unilateral retrograde transpubic screws  9 bilateral retrograde transpubic screws  3 antegrade transpubic screws  1 external fixator | 32 bilateral spinopelvic fixations  4 unilateral triangular spinopelvic fixations  1 bilateral iliac plate fixation (lateral window)  12 unilateral iliac plate fixations (lateral window) | 45 plate osteosyntheses (modified Stoppa approach) |
|  |  | Additional to open reduction procedures | |
|  |  | Posterior pelvic ring | Anterior pelvic ring |
|  |  | 15 in screw augmented transiliosacral screws  2 transiliosacral screws without augmentation | 8 unilateral retrograde transpubic screws  1 bilateral retrograde transpubic screws |
