# Supplementary material for: Percutaneous operative treatment of fragility fractures of the pelvis may not increase the general rate of complications compared to non-operative treatment
Source: Eur J Trauma Emerg Surg. 2021 Apr 3;48(5):3729–35. doi: 10.1007/s00068-021-01660-w (PMC9532300; doi:10.1007/s00068-021-01660-w)
Supplement: Supplementary file 2 — Supplementary file2 (DOCX 27 KB) [file 68_2021_1660_MOESM2_ESM.docx]

**Supplemental table 2** Detailed data overview with regard to fracture classification and therapy of the different fracture types. LOS = length of hospital stay.

| **Classification**  [n (%)] | **Age**  [years mean±SD] | **Gender**  [% female] | **Comorbidities** [%] |  | **Treatment** | **[%]** | **Complications** without UTI [%] | **operative complications** [%] | **LOS** after surgery [days±SD] | **LOS**  [days±SD] | **in hospital mortality** [%] |
| --- | --- | --- | --- | --- | --- | --- | --- | --- | --- | --- | --- |
|  |  |  |  |  |  |  |  |  |  |  |  |
| **FFP Ia** | 82.5 ± 7.8 | 78.6 | *none* | *7.1* | non-operative | 94.6 | 9.4 | - | - | 8.1 ± 4.6 | 1.9 |
| 56 (14.8) |  |  | *≤ 2* | *35.7* | percutaneous | 1.8 | 0 | 0 | 9.0 | 17.0 | 0 |
|  |  |  | *> 2* | *57.1* | ORIF | 3.6 | 0 | 0 | 6.0 ± 2.8 | 11.0 ± 7.1 | 0 |
|  |  |  |  |  |  |  |  |  |  |  |  |
| **FFP Ib** | - | - | - | - | - | - | - | - | - | - | - |
| 0 (0) |  |  |  |  |  |  |  |  |  |  |  |
|  |  |  |  |  |  |  |  |  |  |  |  |
| **FFP IIa** | 80.9 ± 6.5 | 94.1 | *none* | 0 | non-operative | 94.1 | 6.3 | - | - | 8.3 ± 5.3 | 6.3 |
| 17 (4.5) |  |  | *≤ 2* | 35.3 | percutaneous | 0 | 0 | 0 | - | - | 0 |
|  |  |  | *> 2* | 64.7 | ORIF | 5.9 | 0 | 0 | 13.0 | 14.0 | 0 |
|  |  |  |  |  |  |  |  |  |  |  |  |
| **FFP IIb** | 82.9 ± 7.9 | 88.9 | *none* | 1.6 | non-operative | 71.4 | 10.0 | - | - | 8.1 ± 4.8 | 2.2 |
| 126 (33.2) |  |  | *≤ 2* | 42.4 | percutaneous | 19.8 | 4.0 | 8.0 | 9.3 ± 5.4 | 16.7 ± 8.3 | 0 |
|  |  |  | *> 2* | 56.0 | ORIF | 8.7 | 0 | 0 | 9.8 ± 4.0 | 15.5 ± 5.0 | 0 |
|  |  |  |  |  |  |  |  |  |  |  |  |
| **FFP IIc** | 79.6 ± 6.7 | 68.4 | *none* | 5.3 | non-operative | 42.1 | 8.3 | - | - | 9.4 ± 6.3 | 0 |
| 57 (15.0) |  |  | *≤ 2* | 33.3 | percutaneous | 38.6 | 4.5 | 9.1 | 9.5 ± 7.3 | 14.3 ± 9.0 | 0 |
|  |  |  | *> 2* | 61.4 | ORIF | 19.3 | 9.1 | 0 | 11.1 ± 5.7 | 16.4 ± 5.4 | 9.1 |
|  |  |  |  |  |  |  |  |  |  |  |  |
| **FFP IIIa** | 79.2 ± 7.0 | 61.1 | *none* | 0 | non-operative | 25.0 | 11.1 | - | - | 13.3 ± 14.0 | 11.1 |
| 36 (9.5) |  |  | *≤ 2* | 34.3 | percutaneous | 5.6 | 0 | 0 | 11.0 ± 8.5 | 13.5 ± 4.9 | 0 |
|  |  |  | *> 2* | 65.7 | ORIF | 69.4 | 8.0 | 24.0 | 16.3 ± 13.8 | 20.4 ± 15.2 | 4.0 |
|  |  |  |  |  |  |  |  |  |  |  |  |
| **FFP IIIb** | 82.0 ± 9.9 | 100 | *none* | 0 | non-operative | 0 | - | - | - | - | - |
| 2 (0.5) |  |  | *≤ 2* | 50.0 | percutaneous | 0 | - | - | - | - | - |
|  |  |  | *> 2* | 50.0 | ORIF | 100 | 50.0 | 50.0 | 20.5 ± 16.3 | 31.5 ± 12.0 | 50.0 |
|  |  |  |  |  |  |  |  |  |  |  |  |
| **FFP IIIc** | 80.9 ± 7.3 | 95.0 | *none* | 0 | non-operative | 35.0 | 42.9 | - | - | 18.1 ± 22.7 | 0 |
| 20 (5.3) |  |  | *≤ 2* | 40.0 | percutaneous | 45.0 | 0 | 22.2 | 9.1 ± 4.6 | 15.6 ± 3.8 | 0 |
|  |  |  | *> 2* | 60.0 | ORIF | 20.0 | 25.0 | 25.0 | 9.8 ± 2.9 | 16.0 ± 4.5 | 25.0 |
|  |  |  |  |  |  |  |  |  |  |  |  |
| **FFP IVa** | 80.0 ± 9.9 | 0 | *none* | 0 | non-operative | 0 | - | - | - | - | - |
| 2 (0.5) |  |  | *≤ 2* | 0 | percutaneous | 50.0 | 0 | 0 | 7.0 | 14.0 | 0 |
|  |  |  | *> 2* | 100 | ORIF | 50.0 | 100 | 0 | 12.0 | 16.0 | 0 |
|  |  |  |  |  |  |  |  |  |  |  |  |
| **FFP IVb** | 79.8 ± 6.5 | 83.6 | *none* | 0 | non-operative | 18.2 | 10.0 | - | - | 9.6 ± 8.0 | 10.0 |
| 55 (14.5) |  |  | *≤ 2* | 32.7 | percutaneous | 27.3 | 6.7 | 6.7 | 10.9 ± 6.2 | 20.5 ± 9.0 | 0 |
|  |  |  | *> 2* | 67.3 | ORIF | 54.5 | 13.3 | 16.7 | 12.7 ± 8.9 | 18.1 ± 9.3 | 3.3 |
|  |  |  |  |  |  |  |  |  |  |  |  |
| **FFP IVc** | 79.6 ± 8.6 | 62.5 | *none* | 0 | non-operative | 25.0 | 0 | - | - | 8.5 ± 2.1 | 0 |
| 8 (2.1) |  |  | *≤ 2* | 50.0 | percutaneous | 0 | - | - | - | - | - |
|  |  |  | *> 2* | 50.0 | ORIF | 75.0 | 16.7 | 66.7 | 32.3 ± 27.6 | 37.3 ± 26.9 | 0 |
